# Effects of G/A polymorphism, rs266882, in the androgen response element 1 of the *PSA* gene on prostate cancer risk, survival and circulating PSA levels

**DOI:** 10.1038/sj.bjc.6604690

**Published:** 2008-09-30

**Authors:** C Jesser, L Mucci, D Farmer, C Moon, H Li, J M Gaziano, M Stampfer, J Ma, P Kantoff

**Affiliations:** 1 Department of Epidemiology, Harvard School of Public Health, Boston, MA, USA; 2 Channing Laboratory, Brigham and Women's Hospital, Boston, MA, USA; 3 Department of Medicine, Harvard Medical School, Boston, MA, USA; 4 Division of Solid Tumor Oncology, Lank Center for Genitourinary Oncology, Dana-Farber Cancer Institute, Boston, MA, USA; 5 Division of Aging, Brigham and Women's Hospital, Boston, MA, USA

**Keywords:** prostate cancer, PSA gene, ARE1 −158 G/A polymorphism (rs266882), androgen receptor, Physicians' Health Study

## Abstract

Prostate-specific antigen (PSA) is a protease produced in the prostate that cleaves insulin-like growth factor binding protein-3 and other proteins. Production is mediated by the androgen receptor (AR) binding to the androgen response elements (ARE) in the promoter region of the *PSA* gene. Studies of a single nucleotide polymorphism (*PSA* −158 G/A, rs266882) in ARE1 of the *PSA* gene have been conflicting for risk of prostate cancer and effect on plasma PSA levels. In this nested case–control analysis of 500 white cases and 676 age- and smoking-matched white controls in the Physicians' Health Study we evaluated the association of rs266882 with risk and survival of prostate cancer and prediagnostic total and free PSA plasma levels, alone or in combination with *AR* CAG repeats. We used conditional logistic regression, linear regression and Cox regression, and found no significant associations between rs266882 (GG allele *vs* AA allele) and overall prostate cancer risk (RR=1.21, 95% confidence intervals (CI): 0.88–1.67) or prostate cancer-specific survival (RR=0.94, 95%CI: 0.56–1.58). Similarly, no associations were found among high grade or advanced stage tumours, or by calendar year of diagnosis. There was no significant association between rs266882 and baseline total or free PSA levels or the *AR* CAG repeats, nor any interaction associated with prostate cancer risk. Meta-analysis of 12 studies of rs266882 and overall prostate cancer risk was null.

Prostate-specific antigen (PSA) is a protease produced primarily in the prostate and has widely been used as a diagnostic marker of prostate cancer since the early 1990s (Cohen *et al*, 1994). Production of PSA is mediated through binding of the androgen receptor (AR) to androgen response elements (ARE) in the promoter region of the *PSA* gene (Janne *et al*, 1993). Given the importance of PSA as a prostate cancer biomarker, regulation of the gene is of great interest.

A single nucleotide polymorphism in ARE1 (*PSA* −158 G/A) of the *PSA* gene (rs266882), identified in 1999 (Rao and Cramer, 1999), has been the focus of numerous studies of prostate cancer risk. ARE1 is the primary binding site in the promoter of the *PSA* gene, and the A to G substitution of rs266882 may affect the binding affinity of the AR and subsequently affect transcript levels of the PSA gene.

An initial case–control study found that men with the homozygous variant GG genotype were at increased risk of advanced prostate cancer (Xue *et al*, 2000). Subsequent studies on this polymorphism have been conflicting, however, with some reporting an increased risk of prostate cancer or higher PSA levels associated with the G allele (Chiang *et al*, 2004; Binnie *et al*, 2005; Schatzl *et al*, 2005), increased risk with the A allele (Gsur *et al*, 2002; Medeiros *et al*, 2002; Lai *et al*, 2007) or no association with risk (Xu *et al*, 2002; Rao *et al*, 2003; Wang *et al*, 2003; Severi *et al*, 2006; Sieh *et al*, 2006). Among healthy men, the AA genotype was associated with higher serum PSA levels compared with those carrying a G allele (Xue *et al*, 2001). Moreover, the effect of the variant differed as a function of CAG repeat length in the *AR* gene (Xue *et al*, 2001). However, the finding with the G/A variant and PSA levels has not been confirmed (Severi *et al*, 2006). The one study that examined the polymorphism with prostate cancer survival found no significant association with cancer-specific mortality (Severi *et al*, 2006).

To address the disparity of findings of this SNP, we performed a large nested case–control study in the Physicians' Health Study (PHS) to evaluate the rs266882 polymorphism in prostate cancer. We examined associations with risk, survival and prediagnostic baseline plasma PSA levels, alone and in combination with *AR* CAG repeats. Additionally, we performed a meta-analysis of published studies that have examined the rs266882 polymorphism and overall prostate cancer risk.

## Materials and methods

### Study population

The PHS is a randomised double-blind trial of aspirin and *β*-carotene among 22 071 US male physicians, aged 40–84 years in 1982; none had a cancer diagnosis at baseline (1989). The current study was nested among the subset of 14 916 participants, who provided plasma and whole blood prior to randomisation. Men were followed for incident cancer, including prostate, and all cases are confirmed through medical record review. Men with prostate cancer are followed to collect detailed information on disease progression and development of metastases. Cause of death is confirmed by review of death certificate and pertinent medical records. Collection of additional study characteristics including age at enrolment, blood collection and processing, stage classification and clinical follow-up were described earlier (Gann *et al*, 1996).

We included in the study 500 cases diagnosed through 1995. Cases were matched (1 : 1, 1 : 2 or 1 : 3) on age (±1 year when feasible, but up to ±5 years for older men) and smoking status (current, former and never) to controls selected at random from among men free from diagnosed prostate cancer at the time the case was diagnosed. A total of 676 controls were included. All men provided informed consent, and the protocol was approved by the Institutional Review Board at Partners Healthcare.

### Laboratory analysis

Using baseline plasma samples, we measured total and free PSA levels by a double-antibody radioimmmunoassay using rabbit polyclonal anti-PSA on the basis of competitive binding as described earlier (Gann *et al*, 1995). The rs266882 polymorphism was assayed on extracted DNA according to the protocol described by Xue *et al* (2000). Briefly, the alleles of the G/A polymorphism at position −158 in the promoter region of the *PSA* gene were amplified and the three possible genotypes were distinguished by cutting with the *Nhe*I restriction enzyme. The *AR* gene CAG repeat length was determined by running the PCR-amplified fragments on a denaturing polyacrylamide gel with automated fluorescence detection of the fragments and sizing by Genescan (Giovannucci *et al*, 1997).

### Statistical analysis

To limit the potential for population stratification, we restricted the analysis to white men (94% of PHS cohort is white). Hardy–Weinberg equilibrium of allelic frequency was tested by a goodness of fit *χ*^2^-test. We calculated odds ratios as an estimate of relative risk ratios and 95% confidence intervals (CI) using conditional logistic regression, matched on age and smoking status, to evaluate the association between the rs266882 polymorphism and prostate cancer risk. We stratified the analysis further according to tumour grade (low grade: Gleason 2–6 or well differentiated; moderate grade: Gleason 7 or moderately differentiated; high grade: Gleason 8–10 or poorly differentiated), stage (T1/T2 or T3/T4) and calendar year of diagnosis (1982–1992 or 1992–1995). The following number of cases and matched controls (cases; controls) for each subgroup: low grade (265; 360), moderate grade (137; 180), high grade (83; 113), localized: T1/T2 (303; 409), or advanced: T3/T4 (156; 214) tumour stage, diagnosed 1982–1992 (357; 530) and diagnosed 1992–1995 (143; 146).

Additionally, we assessed whether the association of rs266882 genotype and prostate cancer risk differed according to categorical pre-diagnostic PSA levels (total PSA: <4 ng ml^−1^, ⩾4 ng ml^−1^; free PSA: <15%, 15–24%, ⩾25%) or number of AR CAG repeats (tertiles, 7–20, 21–23 and 24–39) using likelihood ratio tests.

We used time to event analyses to evaluate the rs266882 polymorphism and cancer-specific survival. Person–time was calculated from date of cancer diagnosis to prostate cancer death or censored at time of death from other causes or end of follow-up (31 March 2007). We used Cox proportional hazard models to calculate hazard ratios and 95% CI, adjusted for aggressive disease (i.e., stage T3/T4 or high grade), age at diagnosis and date of diagnosis (pre/post 1992).

Linear regression was used to estimate the association among PSA levels (natural log transformed) with rs266882 genotype, separately for cases and controls. An interaction with *AR* CAG repeats was evaluated to determine if the effect of rs266882 genotype differed according to *AR* CAG repeat length. The SAS Statistical Software (Version 9.1) was used for these analyses.

We conducted a meta-analysis of 12 published studies that have evaluated the association between the rs266882 polymorphism and prostate cancer risk (Xue *et al*, 2000; Gsur *et al*, 2002; Medeiros *et al*, 2002; Wang *et al*, 2003; Chiang *et al*, 2004; Binnie *et al*, 2005; Cicek *et al*, 2005; Salinas *et al*, 2005; Severi *et al*, 2006; Sieh *et al*, 2006; Lai *et al*, 2007). To standardise comparisons across the studies, we calculated odds ratios and overall prostate cancer risk comparing the rs266882 GG *vs* AA genotypes and GA *vs* AA genotypes. Inverse variance weighting with a random effects model was used to create a summary estimate using the statistical package Stata (StataCorp, 2003).

## Results

Genotype data was available for 500 cases and 676 controls. Among cases, mean age at diagnosis was 68 years, 17% had poorly differentiated tumours (3% missing) and 31% were advanced tumour stage at diagnosis (8% missing) (Table 1). The majority of cases (71%) were diagnosed before 1992, prior to widespread PSA screening.

The rs266882 genotype distribution among controls (GG: 25%, GA: 47% and AA: 28%) was similar to other studies of white men (Xue *et al*, 2000; Cicek *et al*, 2005; Salinas *et al*, 2005; Sieh *et al*, 2006) and was in Hardy–Weinberg equilibrium (*P*=0.18). Controlling for matching factors, there was no evidence of an association between *PSA* genotype and total prostate cancer risk. Moreover, we found no association between the rs266882 genotype and cancer risk when we stratified within tumour grade or stage, or among those diagnosed pre/post PSA era (Table 2).

As shown earlier in Gann *et al* (2002), pre-diagnostic total and free PSA levels measured in baseline blood were significantly higher among cases compared with controls (Table 3). Using age-adjusted linear regression, we found no significant association between the rs266882 genotype and levels of total or free PSA among either cases or controls. Similarly, we did not see a difference in total prostate cancer risk when the analysis was stratified by rs266882 genotype and total plasma PSA level (*P* for interaction ∼0.41) or free PSA level (*P* for interaction ∼0.70).

An earlier study within the PHS cohort examined a trinucleotide CAG repeat polymorphism in *AR*. Shorter *AR* CAG repeats, which are associated with greater transactivation of the *AR*, were associated with higher risk of advanced prostate cancer compared with longer *AR* CAG repeats (Giovannucci *et al*, 1997). In this study, we examined whether the association between the rs266882 genotype and prostate cancer risk or baseline PSA levels was modified by *AR* CAG repeat length. However, there was no evidence of association between the rs266882 genotype and total prostate cancer risk among men with long or short CAG repeats. In addition, there was no evidence of modification by *AR* CAG repeat length on associations between the rs266882 genotype on total plasma PSA levels (*P*-value for interaction: controls, *P*=0.80; cases, *P*=0.25) and free PSA levels (controls, *P*=0.30; cases, *P*=0.96).

Among the 500 men with prostate cancer, 111 men died of their disease. The median survival time between diagnosis and prostate cancer-specific death was 13.5 years (range: <1–24.3 years). We found no association between the rs266882 genotype and prostate cancer survival (Table 4).

**Figure 1:**
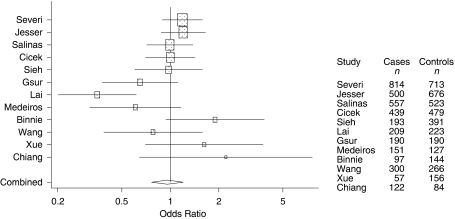
Meta-analysis of 12 studies of the association between rs266882 GG genotype compared with AA genotype and overall prostate cancer risk. Individual and random effects, summary odds ratios and 95% confidence intervals (CI). The shaded squares and horizontal lines indicate the study-specific odds ratio and 95% CI for the rs266882 GG genotype compared with the AA genotype. The area of the shaded square is proportional to the inverse of the sum of the between studies variance and the study-specific variance. The diamond is the summary odds ratio and 95% CI.

Figure 1 presents the estimates from each of the 12 studies of the rs266882 genotype and prostate cancer risk, and the summary odds ratios from the meta-analysis. There was no evidence of an association between the GG (OR=0.96, 95% CI: 0.77, 1.20) (Figure 1) or GA (OR=0.92, 95% CI: 0.82, 1.03) genotypes compared with AA. Sensitivity analyses were performed by restricting the meta-analysis to eight studies conducted among predominantly white populations that reported similar rs266882 genotype distribution among controls. These analyses produced results similar to the full meta-analysis of 12 studies.

## Discussion

In this large prospective study, we comprehensively assessed the relation of the ARE1 (*PSA* −158 G/A) polymorphism (rs266882 genotype) and prostate cancer risk, survival and pre-diagnostic plasma PSA levels. We found no association between the *PSA* polymorphism and cancer risk, overall or by grade, stage or calendar year of diagnosis. Similarly, the rs266882 genotype was not associated with cancer-specific survival. Finally, there was no association between the rs266882 genotype and PSA plasma levels among cases or controls. There were no significant interactions of the rs266882 genotype with PSA plasma level or *AR* CAG repeat length for any of the outcomes examined.

Given the importance of PSA, regulation of its production is of biological and clinical importance. For example, PSA cleaves insulin-like growth factor binding proteins resulting in local release of IGF-1 (Cohen *et al*, 1994), which has been positively associated with prostate cancer risk (Chan *et al*, 1998; Stattin *et al*, 2000). PSA has also been demonstrated to activate the small latent form of transforming growth factor-*β*2 (TGF-*β*2) (Dallas *et al*, 2005), one of the isoforms of TGF-*β*, a potent growth factor that acts as a tumour enhancer once cancer cells have become refractory to its tumour suppressor effects (Massague, 2008). Activation of TGF-*β*2 by PSA may potentially contribute to the formation of osteoblastic lesions in bone metastatic prostate cancer (Dallas *et al*, 2005).

Accordingly, numerous investigators have examined the function of the rs266882 polymorphism in PSA regulation alone or in combination with other genes, with contradictory results. An initial relatively small case–control study of non-Hispanic white men (57 cases, 156 controls) reported a positive association of the GG allele of rs266882 with a three-fold risk of advanced cancer (Xue *et al*, 2000). Subsequent studies also reported increased risk of prostate cancer with the G allele in Taiwan (122 cases, 84 controls)(Chiang *et al*, 2004) and Scotland (97 cases, 144 controls)(Binnie *et al*, 2005). A larger sibling-based case–control study (439 cases, 479 controls) in a predominantly white American population found the GG allele associated with risk of non-aggressive disease (Cicek *et al*, 2005). In contrast, a large case–control study of 99% Caucasian Australians (821 cases, 734 controls) found a significant association for the G allele with stage III to IV tumours, but not for overall prostate cancer (Severi *et al*, 2006).

Conversely, other studies found an association between the A allele and increased risk of prostate cancer (Gsur *et al*, 2002; Medeiros *et al*, 2002; Lai *et al*, 2007). A case control study of Portuguese men (151 cases, 127 controls) found a three-fold increased risk for men less than 67 years with the AA allele (Medeiros *et al*, 2002). Similarly, a larger study of Australian Caucasian men (209 cases, 223 controls) found a three-fold risk of prostate cancer with the AA allele (Lai *et al*, 2007).

Several studies, including one in Japanese men (300 cases, 216 BPH controls, 266 controls) (Wang *et al*, 2003) and a predominantly (∼95%) white American population (591 cases, 538 controls) (Salinas *et al*, 2005), as well as the present study, found no significant association between the *PSA* polymorphism and risk of prostate cancer. Although, our own study cannot rule out small relative risks, taken together with our meta-analysis of the 12 studies suggests that there is no overall effect of the variant on prostate cancer risk.

Only one published study has examined the rs266882 variant with survival of prostate cancer (Severi *et al*, 2006). The average follow-up was 8.2 years, with 68 deaths. Our study was consistent with the Severi *et al* (2006) study in finding no significant association with the rs266882 genotype and risk of dying from prostate cancer.

It has been hypothesised that the rs266882 polymorphism may be associated with serum levels of PSA through higher binding affinity of the ARE1 with either the A or G allele. Results of such studies also have been contradictory. Among healthy controls (PSA levels <4 ng ml^−1^), higher serum PSA levels were statistically significantly associated with AA genotype when all ethnic groups were combined, but not for any of the ethnic groups individually, suggesting population stratification may be a possibility for this finding (Xue *et al*, 2001). In another study of white men with PSA levels <4 ng ml^−1^, men with the AA genotype had a 28% higher level of PSA than men with the GG genotype (Salinas *et al*, 2005). Two studies of healthy controls with PSA levels <4 ng ml^−1^ (Rao and Cramer, 1999; Wang *et al*, 2003) and one with PSA <9 ng ml^−1^ (Severi *et al*, 2006) reported no association of this genotype with serum PSA level. These null results were consistent with our findings.

*AR* regulates PSA expression by binding androgen response elements. Some studies of *AR*, including ours, found shorter CAG repeats associated with increased expression of AR and with higher risk of advanced prostate cancer compared with longer CAG repeats (Giovannucci *et al*, 1997). Therefore, the potential interaction of the rs266882 genotype with CAG repeats in the *AR* is of interest. Xue *et al* (2000) reported a five-fold increased risk for prostate cancer among white men with both a short CAG allele and PSA genotype GG. In the full multi-ethnic cohort, Xue *et al* (2001) also reported a significant interaction (0.049) with serum PSA, which increased 7% with each decrease of one CAG among those with the AA genotype for the *PSA* gene. Models were adjusted for age and ethnicity, however residual confounding by ethnicity in this very diverse cohort could account for this borderline significant finding. Four additional studies, as well as the present, found no interaction between rs266882 genotype and *AR* CAG length in relation to plasma PSA levels (Xu *et al*, 2002; Rao *et al*, 2003; Salinas *et al*, 2005; Sieh *et al*, 2006).

The results of this large study with long-term and prospective follow-up, taken together with earlier findings and the results of the meta-analysis, add further support for the conclusion that the rs266882 polymorphism is unrelated to prostate cancer risk, survival or to plasma PSA levels.

## Figures and Tables

**Table 1:** Characteristics of prostate cancer cases in the Physicians' Health Study, incidence 1982–1995 and survival 1982–2007

	**Cases (*N*=500)**
Mean age at baseline (±s.d.)	61.2 (7.6)
Mean age at diagnosis (±s.d.)	68.4 (6.8)
	
*Grade at diagnosis* (%)^a^,b	
Low grade	265 (53.0)
Moderate grade	137 (27.4)
High grade	83 (16.6)
	
*Tumour stage at diagnosis* (%)^c^	
T1/T2	303 (60.6)
T3/T4	156 (31.2)
	
*PSA era* (%)	
Before 1992	357 (71.4)
1992 or after	143 (28.6)
	
*Died from prostate cancer* (%)	
Died	111 (22.2)
Survived	389 (77.8)

PSA=Prostate-specific antigen.

a 15 cases missing grade.

b Low-grade disease: Gleason 2–6 or well differentiated; Moderate grade: Gleason 7 or moderately differentiated; High grade: Gleason 8–10 or poorly differentiated.

c 41 cases missing stage.

**Table 2:** Odds ratios^a^ and 95% confidence intervals of association between ARE1 (*PSA* −158 G/A) genotype and prostate cancer risk, overall and by tumour grade,^b^ stage and calendar year of diagnosis

		***PSA* genotype**		
	***N* (cases/controls)**	**AA**	**GA**	**GG**
Total prostate cancer	500/676	1.00 (Ref)	1.00 (0.76–1.33)	1.21 (0.88–1.67)
				
*Tumour grade* b				
Low grade	265/360	1.00 (Ref)	0.88 (0.61–1.29)	1.25 (0.81–1.93)
Moderate grade	137/180	1.00 (Ref)	1.11 (0.63–1.95)	1.41 (0.75–2.65)
High grade	83/113	1.00 (Ref)	1.39 (0.63–3.08)	1.22 (0.51–2.90)
				
*Tumour stage*				
T1/T2	303/409	1.00 (Ref)	1.01 (0.71–1.46)	1.33 (0.89–1.99)
T3/T4	156/214	1.00 (Ref)	0.90 (0.53–1.51)	0.86 (0.45–1.62)
				
*Diagnosis date*				
Before 1992	357/530	1.00 (Ref)	0.94 (0.67–1.31)	1.05 (0.72–1.55)
1992 or after	143/146	1.00 (Ref)	1.14 (0.67–1.94)	1.69 (0.92–3.08)

PSA=Prostate-specific antigen.

Physicians' Health Study, 1982–1995.

a Odds ratios are controlled for age and smoking by matching.

b Low-grade disease: Gleason 2–6 or well differentiated; Moderate grade: Gleason 7 or moderately differentiated; High grade: Gleason 8–10 or poorly differentiated.

**Table 3:** Median and 25th, 75th percentile (IQR) distributions of baseline total and free PSA levels among prostate cancer cases and controls according to *PSA* ARE1 −158 G/A genotype^a^

		**Total PSA**	**Free PSA**
***PSA* genotype**	***n* b**	**Median (IQR)**	**Median (IQR)**
*Cases*			
AA	83	2.4 (1.3, 5.0)	0.4 (0.3, 0.7)
GA	159	2.7 (1.5, 5.6)	0.5 (0.3, 0.8)
GG	93	2.7 (1.6, 5.9)	0.5 (0.3, 0.9)
			
*Controls*			
AA	172	1.1 (0.7, 1.9)	0.3 (0.2, 0.4)
GA	278	1.1 (0.7, 2.0)	0.3 (0.2, 0.5)
GG	139	1.0 (0.6, 2.1)	0.3 (0.2, 0.5)

PSA=Prostate-specific antigen.

Physicians' Health Study, 1982–1995.

a Age-adjusted estimates for effect of *PSA* genotypes on total or free PSA from linear regression models were all non-significant at the *α*=0.05 level.

b Number of cases and controls were the same for total and free PSA with the exception of cases/GA allele/free PSA, *n*=158.

**Table 4:** Hazard ratios and 95% confidence intervals of ARE1 (*PSA* −158 G/A) genotype and prostate cancer-specific survival, overall and stratified by grade^a^, stage and calendar year of diagnosis

	***n***		***PSA* genotype**		
	**Cases**	**Deaths**	**AA**	**GA**	**GG**
Overall survival^b^	500	111	1.00 (Ref)	0.96 (0.61–1.51)	0.94 (0.56–1.58)
					
*Tumour grade* c					
Low grade	265	29	1.00 (Ref)	0.59 (0.26–1.33)	0.39 (0.14–1.10)
Moderate grade	137	32	1.00 (Ref)	0.97 (0.43–2.21)	0.73 (0.27–1.96)
High grade	83	43	1.00 (Ref)	1.42 (0.57–3.53)	1.81 (0.70–4.74)
					
*Tumour stage* c					
T1/T2	303	37	1.00 (Ref)	0.81 (0.38–1.72)	0.67 (0.27–1.65)
T3/T4	156	68	1.00 (Ref)	1.13 (0.62–2.05)	1.07 (0.54–2.11)

PSA=Prostate-specific antigen.

Physicians' Health Study, 1982–2007.

a Low-grade disease: Gleason 2–6 or well differentiated; Moderate grade: Gleason 7 or moderately differentiated; High grade: Gleason 8–10 or poorly differentiated.

b Hazard ratios controlled for aggressive disease (i.e., stage T3/T4 or high grade), age at diagnosis and date of diagnosis (pre/post 1992).

c Hazard ratios controlled for age at diagnosis and date of diagnosis (pre/post 1992).
